# Do Confidence Ratings Reactively Modify Children’s Academic Assessment Performance? Negative Answer from a Three-Year Longitudinal Study

**DOI:** 10.3390/jintelligence12090091

**Published:** 2024-09-23

**Authors:** Jun Zheng, Ningxin Su, Tian Fan, Baike Li, Wenbo Zhao, Xiao Hu, Chunliang Yang, Liang Luo

**Affiliations:** 1 Center for Teacher Education Research, Faculty of Education, Beijing Normal University, Beijing 100875, China; zhengjunpsy@mail.bnu.edu.cn; 2 Joint Education Institute of Zhejiang Normal University and University of Kansas, Zhejiang Normal University, Jinhua 321004, China; suningxin@mail.bnu.edu.cn; 3 Collaborative Innovation Center of Assessment for Basic Education Quality, Beijing Normal University, Beijing 100875, China; fantian@mail.bnu.edu.cn; 4 School of Psychology, Liaoning Normal University, Dalian 116029, China; baikeli94@gmail.com; 5 School of Sociology, Beijing Normal University, Beijing 100875, China; zhaowb@mail.bnu.edu.cn; 6 Beijing Key Laboratory of Applied Experimental Psychology, National Demonstration Center for Experimental Psychology Education, Faculty of Psychology, Beijing Normal University, Beijing 100875, China; bnuhx2010@foxmail.com; 7 Institute of Developmental Psychology, Faculty of Psychology, Beijing Normal University, Beijing 100875, China; 8 State Key Laboratory of Cognitive Neuroscience and Learning, Beijing Normal University, Beijing 100875, China

**Keywords:** confidence ratings, academic assessment performance, reactivity, longitudinal study, self-confidence

## Abstract

The reactivity effect of metacognitive judgments on first-order task performance has received increased research attention. Previous studies showed that soliciting retrospective confidence ratings (CRs) reactively enhances task performance itself, such as performance in decision making and reasoning tasks, especially for those with high self-confidence. It remains unknown whether CRs can improve students’ academic assessment performance in real educational settings. The current study recruited 795 fourth-grade elementary school children to explore if making CRs reactively affects students’ academic assessment performance in two main subjects (i.e., Chinese Language and Mathematics). The data were collected across six waves with half-year intervals. From Wave 2, children either provided (CR group) or did not provide CRs (no-CR group) when completing standardized academic assessments. The results showed Bayesian evidence supporting the claim that making CRs does not influence children’s academic assessment performance (both the average performance across waves 2–6 and the performance in each wave) in both subjects. Furthermore, children’s self-confidence did not moderate the reactive influence of CRs. The results from multilevel regression analyses re-confirmed the above conclusions. Possible explanations for the absence of the reactivity effect of CRs on children’s academic assessment performance are discussed.

## 1. Introduction

People experience a variety of examinations from an early age, such as taking regular classroom quizzes and performing standardized aptitude tests. At the end of a test, people often retrospectively evaluate their own performance and then use the evaluation results to regulate subsequent learning behaviors, ultimately impacting future performance (Bjork et al. 2013). This is a typical scenario of the long-established indirect effect of metacognitive monitoring on task performance via its influence on metacognitive control (Nelson and Narens 1990). Namely, people typically regulate their subsequent learning behaviors (e.g., study-time allocation, study-strategy regulation) according to the results of metacognitive judgments (e.g., judgments of learning, JOLs; and confidence ratings, CRs), which in turn affect the final learning performance (Ariel et al. 2009; Metcalfe and Kornell 2005; Son and Metcalfe 2000; Tullis et al. 2012). But, if metacognitive judgments are elicited during the testing phase (e.g., making CRs after test takers answering each question), does this on-line (i.e., real-time) monitoring process produce an immediate influence on the concurrent task performance? This is an interesting and promising question, but little research has examined it, especially in real educational settings. Addressing this question involves the recently widely studied phenomenon known as the reactivity effect of metacognitive *judgments* (for reviews, Double et al. 2018; Double and Birney 2019b).

Metacognitive judgments, such as JOLs and CRs, have been widely used to measure individuals’ metacognitive ability (e.g., Fleming and Lau 2014; Rhodes and Castel 2008; Roebers and Spiess 2017). Recent studies established that these metacognitive judgments are not passive measures of metacognition because they can reactively change the way in which a task will be accomplished and then alter task performance. As an illustration of reactivity, many studies have showed that, making item-by-item JOLs during the learning phase can promote memory performance for word lists (e.g., Zhao et al. 2022; Zheng et al. 2024), related word pairs (e.g., Soderstrom et al. 2015; Witherby and Tauber 2017), and visual images (e.g., Shi et al. 2022), whereas making JOLs has a minimal influence on memory for educationally related texts (Ariel et al. 2021; Hausman and Kubik 2023) and general knowledge facts (Schäfer and Undorf 2023) and even lead to small-to-moderate negative reactivity for unrelated pairs (Undorf et al. 2024).

Although a growing body of recent studies has documented a positive reactivity effect of metacognitive judgments on task performance (e.g., decision accuracy, memory recall) for certain materials (e.g., Fox and Charness 2010; Janes et al. 2018; Shi et al. 2022) and certain populations (e.g., Double and Birney 2017; Tauber and Witherby 2019; Zhao et al. 2022), educational implications of the reactivity effect have rarely been tested (Ariel et al. 2021; Schäfer and Undorf 2023), especially for children’s academic assessment performance in real educational settings. Recent evidence suggested that, when participants are asked to report trial-by-trial response confidence following making each decision, their decision accuracy is reactively improved by the requirement of reporting response confidence (e.g., Lei et al. 2020; Li et al. 2023). The positive reactivity effect of CRs on decision accuracy prompts us to suspect that simply instructing students to report CRs while completing an academic test may reactively enhance their academic assessment performance. To the best of our knowledge, no prior research has explored whether soliciting CRs can produce direct benefits to children’s academic assessment performance. Hence, the current study aims to fill this important gap. Below, we briefly review previous findings of the CR reactivity effect and then provide an overview of the current study.

### 1.1. Reactivity of Confidence Ratings

Like prospective JOLs, retrospective CRs can also reactively improve participants’ cognitive performance (e.g., Bonder and Gopher 2019; Double and Birney 2017, 2018, 2019a; Lei et al. 2020; Li et al. 2023). For example, Double and Birney (2017) instructed two groups (a CR group vs. a no-CR group) of participants to complete a 20 min test consisting of 20 items drawn from the Raven’s Advanced Progressive Matrices Task. In the CR group, participants had to rate their confidence (i.e., “*How confident are you in your answer*”) on a 6-point scale after answering each reasoning question. By contrast, in the no-CR group, participants completed the test without providing CRs. The results showed that test scores (i.e., the number of reasoning questions correctly answered) were significantly higher in the CR than in the no-CR group. So far, the positive reactivity effect of CRs has been validated on several types of tasks, including decision making (Bonder and Gopher 2019; Lei et al. 2020; Li et al. 2023) and reasoning tasks (Double and Birney 2017, 2018). Additionally, this effect has also been corroborated in different populations, including older (Double and Birney 2017, 2018) and young adults (Bonder and Gopher 2019; Double and Birney 2017; Lei et al. 2020; Li et al. 2023). However, it remains unknown whether the positive reactivity effect of CRs can generalize to other tasks (e.g., academic tests) and populations (e.g., elementary school children). Given the methodological and educational significance, the current study aims to explore the potential influence of CRs on elementary school children’s academic assessment performance in real educational settings. The theoretical assumptions presented below offer some predictions about the direction of the CR reactivity effect on children’s academic assessment performance.

The *general cognitive benefit* hypothesis proposes that making CRs confers some kind of benefits to performance monitoring, which in turn facilitates the regulation of cognitive processes and then produces a superior task performance (Double and Birney 2017, 2018, 2019a). Specifically, to provide a CR, participants have to reflect on the correctness of their answers, which may facilitate performance monitoring on current and succeeding items. As a result, people may engage in effective monitoring and control processes, such as error detection (Rinne and Mazzocco 2014), strategy regulation (Sahakyan et al. 2004), and decision making about when to progress to the next item (Ackerman 2014), which will in turn improve the final task performance. Thus, this hypothesis predicts a positive CR reactivity effect on children’s academic assessment performance.

The second hypothesis for CR reactivity is the *enhanced conservation* theory, recently proposed by Li et al. (2023). This theory assumes that repeatedly asking participants to report response confidence provokes feelings of uncertainty (i.e., enhancing awareness that their responses may be incorrect), in turn making them more cautious (i.e., more conservative and more careful) to make decisions in the following trials. To provide an appropriate CR for a given decision, people may need to gather more information (or evidence) before making the decision. Indeed, Li et al. (2023) found that soliciting CRs reactively enhanced decision accuracy and slowed down decision speed by improving decision threshold, as estimated by a drift diffusion model (DDM). Accordingly, the enhanced conservation theory predicts that soliciting CRs should reactively enhance children’s academic assessment performance by making them more carefully answer test questions.

Recent findings further suggest that the reactivity effect of CRs tends to be moderated by other factors, such as individuals’ initial level of self-confidence (Double and Birney 2017, 2018, 2019a). For instance, Double and Birney (2018) revealed that older adults’ initial level of self-confidence in their own reasoning abilities moderated the reactivity effect of CRs on reasoning performance. Specifically, making CRs enhanced reasoning performance for participants with high self-confidence but undermined performance for those with low self-confidence. Double and Birney (2018) borrowed the *cognizant confidence* hypothesis to explain their findings: CRs prime pre-existing beliefs about one’s ability and thus generate divergent reactivity effects between individuals with high versus low self-confidence (Double and Birney 2017, 2018, 2019a). The frequent requirement of making CRs may evoke individuals’ awareness of their self-confidence in performing such tasks. Thus, self-evaluation activated by the requirement of making CRs may be affirming for those who believe they are good at a given task (i.e., individuals with high self-confidence), but threatening for those who believe they are poor at that task (i.e., individuals with low self-confidence). Prior studies demonstrated that self-confidence is closely related to task motivation, such as self-concept and self-efficacy (Kröner and Biermann 2007; Stankov et al. 2012), which in turn influences task performance (Honicke and Broadbent 2016). Thus, the cognizant confidence hypothesis predicts that self-confidence may moderate the reactivity effect of CRs on children’s academic assessment performance.

### 1.2. Overview of the Current Study

As noted above, no prior studies have been conducted to explore the reactive influence of CRs on children’s academic assessment performance. Whether children’s initial self-confidence (i.e., prior beliefs about their own academic abilities) moderates the CR reactivity effect also remains unknown. Given that exploring children’s metacognitive abilities requires attention to whether soliciting metacognitive judgments induces reactivity effects and determining how CRs reactively influence children’s learning performance in real educational settings has practical significance, this study endeavors to employ a three-year longitudinal design to develop more insights into these two issues. A total of 795 fourth-grade children were recruited, and their academic performance on Chinese Language and Mathematics, two main subjects for Chinese pupils, was assessed across six waves at half-year intervals. From the second assessment wave, children who were randomly assigned to the CR group completed Chinese Language and Mathematics tests with making CRs, while children in the no-CR group completed the tests without making CRs. The CR reactivity effect on academic assessment performance was quantified as the difference in test performance between the CR and no-CR groups in each subject. The potential moderating effect of self-confidence in CR reactivity was also assessed here.

The purpose of using repeated measurements in the two main subjects is to obtain relatively reliable conclusions. Therefore, we first calculated the average scores of Chinese Language tests and the average scores of Mathematics tests across the last five consecutive waves (i.e., T2–T6, at which times the experimental manipulations were implemented), respectively, and then compared the difference in each subject’s test scores between the CR and no-CR groups. Secondly, we averaged self-confidence in their own Chinese Language and Mathematics academic achievements across T2–T6 (see below for details), and then performed multiple regression analyses to examine the interaction between the experimental groups and self-confidence (i.e., the moderating effect of self-confidence). As supplemented in the Appendix, we also conducted the aforementioned analyses at each time point of T2–T6. Finally, multilevel regression analyses were performed to further refine the analyses for CR reactivity and the moderating effect of self-confidence.

## 2. Method

### 2.1. Participants

Participants were recruited from a local elementary school in Hebei Province, China. The data were collected across six waves, beginning with the children’s fourth grade. All fourth graders at this school were included in the longitudinal project, with a total of 795 children (*M*_age_ = 9.71 years, *SD* = 0.32, 420 boys) from 18 classes. The initial assessment wave (i.e., T1) was administered in December 2016, and subsequent assessments (i.e., T2-T6) were conducted with six-month intervals. At each wave, participants were organized to complete standardized academic tests and questionnaires.

We randomly selected 6 classes of students to be assigned to the experimental group (i.e., the CR group), with the remaining 12 classes serving as the control group (i.e., the no-CR group). Participants did not undergo any experimental manipulations at T1 (i.e., serving as the baseline measurement), but received different experimental manipulations (i.e., with or without making CRs) in the following waves of T2–T6. Specifically, at each wave of T2–T6, all participants completed the standardized Chinese Language and Mathematics achievement tests (Dong and Lin 2011; Lv et al. 2020), with the CR group providing a CR after answering each test question, whereas the no-CR group did not provide any CRs.

At each wave of T1–T6, the participants were 791, 792, 781, 777, 772, and 773 children, respectively. The age and gender of the two groups are shown in Table 1. Attrition across waves was primarily due to children’s sick leave or transfers to other schools. All participants were native Chinese speakers and typically developing children, as reported by the school and parents. Outliers more than 3 *SD*s from the group mean were identified and excluded from data analyses. Specifically, for Chinese Language achievement tests, there were 10 extreme values at T1 (with 6 in the CR group and 4 in the no-CR group), 4 extreme values at T2 (with 2 in the CR group and 2 in the no-CR group), 4 extreme values at T3 (with 2 in the CR group and 2 in the no-CR group), and 1 extreme value at T4 (in the CR group). There were no outliers on Mathematics achievement tests at any waves. Note that all results showed the same patterns regardless of whether the outliers were excluded or not.

The present study was approved by the Ethics Committee of the Collaborative Innovation Center of Assessment toward Basic Education Quality, Beijing Normal University. Informed consent was obtained from the children’s parents by sending a consent letter to each participant’s home to obtain parental approval.

### 2.2. Materials and Procedure

The test questions in each of the standardized Chinese Language achievement tests and Mathematics achievement tests were drawn from the National Development Project (Dong and Lin 2011), which were developed based on the national curriculum standards for elementary school students. The Chinese Language achievement tests assessed knowledge of language and culture as well as reading comprehension (Lv et al. 2020). The Mathematics achievement tests measured contents of numbers and algebra, space and shapes, and statistics and probability (Lv et al. 2020). Of the six waves, the Chinese Language achievement tests consisted of 48, 47, 45, 52, 52, and 52 questions, respectively, all of which were multiple-choice questions. The Mathematics achievement tests consisted of 32, 30, 40, 28, 32, and 28 questions, respectively, including multiple-choice questions and problem-solving questions. The participants’ assessment scores in each test ranged from 0 to 100. In this sample, McDonald’s *omega* (Dunn et al. 2014) of Chinese Language achievement tests ranged from .84 to .92, and those of Mathematics achievement tests ranged from .73 to .86, indicating that all the tests had good reliability.

Participants were assessed during regular classes at school. All tests were implemented in a paper-and-pencil manner, administered under supervision by well-trained research assistants, all of whom were psychology graduate students. The procedure was the same across all six waves, except a minor difference in experimental manipulation. On each testing day, participants completed the Chinese Language achievement test and some questionnaires in the morning, and then completed the Mathematics achievement test and some questionnaires in the afternoon. For both groups, participants were given 45 min to complete each of the Chinese Language and Mathematics tests. At each wave of T2–T6, both groups were given identical tests except for the difference in the requirement of reporting CRs. In the CR group, after answering each test question, participants had to rate their confidence about the correctness of their answer on a scale ranging from 1 (*I’m not confident at all*) to 7 (*I’m very confident*). The scale was presented below each test question and asked, “*How confident are you in your answer*?”. A pilot study established that fourth graders are fully able to understand the requirement of the CR task.

Each student’s self-confidence about their academic achievement was measured at the post-test questionnaire phase. Specifically, participants were asked to estimate how many scores (ranging from 0 to 100) they would gain in the final exams on Chinese Language and Mathematics, respectively. Higher scores indicate greater self-confidence in academic performance in the Chinese Language or Mathematics subject.

## 3. Results

Below, we first report academic assessment performance results (i.e., whether soliciting CRs reactively modifies elementary children’s academic assessment performance). Then, we assess the potential moderating effect of self-confidence (i.e., whether children’s self-confidence in their own academic performance moderates the potential reactivity effect of CRs). The accuracy of item-by-item CRs is not of substantive research interest and hence is reported in Appendix A. All Bayes analyses presented below were performed via JASP 0.16.4, and multilevel regression analyses were conducted via R 2023.12.1.

### 3.1. CR Reactivity on Academic Assessment Performance

**Figure 1:**
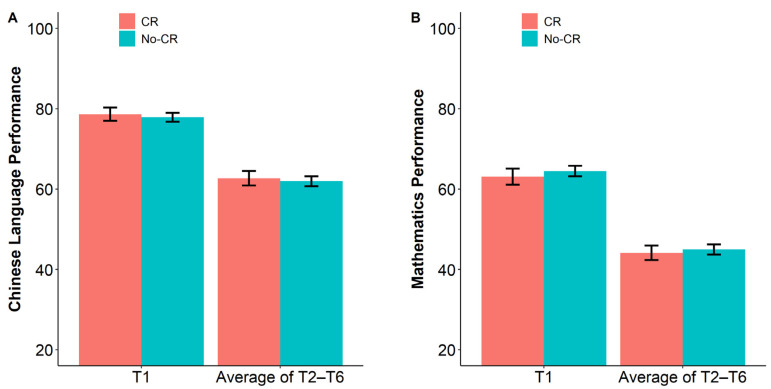
Academic assessment performance as a function of group. (**A**) Chinese Language assessment performance; (**B**) Mathematics assessment performance. Error bars represent 95% CI.

Figure 1 depicts academic assessment performance as a function of group. An independent sample *t*-test was performed to examine whether CRs reactively changes children’s academic assessment performance. Furthermore, a Bayesian analysis was conducted to assess whether the documented findings favor the alternative (*H*_1_; i.e., existence of CR reactivity) over the null hypothesis (*H*_0_; i.e., absence of CR reactivity). The Bayes Factor (*BF*_10_) is the ratio of the likelihood of data fitting the alternative hypothesis relative to the null hypothesis, with *BF*_10_ > 1 providing support for the alternative hypothesis over the null hypothesis and *BF*_10_ < 1 providing support for the null hypothesis over the alternative hypothesis (van Doorn et al. 2021).

First, the T1 academic assessment performance was analyzed. All participants did not provide item-by-item CRs at T1, so performance at this time point was used as the benchmark of the two groups. Analyses showed no detectable difference in Chinese Language scores between the CR (*M* = 78.63, *SD* = 13.44) and no-CR (*M* = 77.86, *SD* = 12.98) groups, *t*(777) = 0.77, *p* = .44, Cohen’s *d* = 0.06, *BF*_10_ = 0.11. Again, there was no detectable difference in Mathematic scores between the CR (*M* = 63.09, *SD* = 16.67) and no-CR (*M* = 64.46, *SD* = 15.27) groups, *t*(788) = −1.16, *p* = .25, Cohen’s *d* = −0.09, *BF*_10_ = 0.16. Overall, T1 results show Bayesian evidence supporting no baseline difference in Chinese Language and Mathematics academic performance between the two groups.

Then, the average scores of Chinese Language tests and the average scores of Mathematics tests across T2 to T6 were calculated, respectively. The results showed little difference in the average Chinese Language scores between the CR (*M* = 62.69, *SD* = 14.85) and no-CR (*M* = 61.95, *SD* = 14.07) groups, *t*(792) = 0.69, *p* = .49, Cohen’s *d* = 0.05, *BF*_10_ = 0.11. Similarly, the difference in the average Mathematics scores between the CR (*M* = 44.13, *SD* = 15.08) and no-CR (*M* = 44.97, *SD* = 14.55) groups could also be considered negligible, *t*(792) = −0.77, *p* = .44, Cohen’s *d* = −0.06, *BF*_10_ = 0.11. These results show Bayesian evidence that soliciting CRs produces little reactive impact on children’s academic assessment performance in both Chinese Language and Mathematics academic assessments, i.e., there is no reactivity effect of CRs on children’s academic assessment performance in the Chinese Language and Mathematics subjects.

We also analyzed the data for each wave of T2–T6. The results consistently showed no statistically detectable differences between the two groups at each wave (see Table A1 for detailed results). In addition, in order to balance the sample size between the two groups, we conducted supplementary analyses by randomly selecting a set of six classes from the no-CR group. The results again showed no detectable difference in test scores between the CR and no-CR groups in both the Chinese Language and Mathematics tests, all *p*s > .05.

### 3.2. Moderating Effect of Self-Confidence

Multiple regression analyses were conducted on Chinese Language and Mathematics scores, respectively, with group, self-confidence, and their products as predictors. Self-confidence at each wave was mean-centered, and the experimental group was dummy coded (CR group = 1, no-CR group = 0). Furthermore, Bayesian regressions were implemented with a uniform prior model probabilities of 0.20 (Faulkenberry et al. 2020) and the Bayesian inclusion factor (*BF*_incl_) was estimated for every predictor in the model. *BF*_incl_ quantifies the change from prior to posterior inclusion odds of the specific predictor in the model, with *BF*_incl_ > 1 representing evidence supporting inclusion over exclusion of this predictor and *BF*_incl_ < 1 indicating evidence supporting exclusion over inclusion of this predictor.

For each participant, the average of estimates of Chinese Language final exam performance across T2–T6 was calculated as an index of self-confidence in their academic ability in performing Chinese Language exams. The average of estimates of Mathematics final exam performance was also calculated as an index of self-confidence in performing Mathematics exams. In terms of the average Chinese Language assessment scores, the regression model (*R*^2^ = .25, *F*(3,790) = 89.32, *p* < .001) indicated no main effect of group, *β* = 0.32, *t* = 0.34, *p* = .73, *BF*_incl_ = 0.05, re-confirming no reactivity effect of CRs on children’s Chinese Language assessment performance. The average of self-confidence in performing Chinese Language exams positively predicted Chinese Language assessment scores, *β* = 1.02, *t* = 12.04, *p* < .001, *BF*_incl_ = 1.89 × 10^48^. More importantly, there was no interaction between group and self-confidence, *β* = 0.11, *t* = 0.81, *p* = .42, and *BF*_incl_ = 0.03, suggesting a minimal moderating effect of self-confidence on CR reactivity.

In terms of the average Mathematics assessment scores, the regression model (*R*^2^ = .39, *F*(3,790) = 165.98, *p* < .001) showed no main effect of group, *β* = −0.62, *t* = −0.71, *p* = .48, *BF*_incl_ = 0.05, re-confirming no reactivity effect of CRs on children’s Mathematics assessment performance. Self-confidence in performing Mathematics exams positively predicted Mathematics assessment scores, *β* = 1.05, *t* = 17.73, *p* < .001, *BF*_incl_ = 7.73 × 10^81^. Critically, there was no interaction between group and self-confidence, *β* = −0.07, *t* = −0.70, *p* = .49, *BF*_incl_ = 0.02, re-confirming a minimal moderating effect of self-confidence on CR reactivity.

As a conclusion, the Bayesian evidence supports the null hypotheses that soliciting CRs does not affect children’s academic assessment performance, and self-confidence plays no moderating role. It should be noted that the moderating effects of gender were also examined, and the results showed no moderating effect of gender^1^, *p*s > .05.

Further tests were performed to determine the potential moderating role of self-confidence in the reactivity effect of CRs on children’s academic assessment performance. If CRs enhance performance for children with high self-confidence and impair performance for those with low self-confidence (i.e., if making CRs enlarge the difference in assessment performance between children with high and low self-confidence) as predicted by the cognizant confidence hypothesis, then the variance of academic assessment scores should be greater in the CR than in the no-CR group. To test this prediction, Levene’s tests of equality of variances were performed to determine whether the variance of test scores in the CR group was larger than that in the no-CR group. The results showed no detectable difference in the variance of Chinese Language assessment scores between the two groups, *F*(1,792) = 0.86, *p* = .36. Similarly, there was no detectable difference in the variance of average Mathematics assessment scores between the two groups, *F*(1,792) = 0.82, *p* = .37. These results re-confirm that there is no moderating effect of self-confidence.

We also conducted a series of regression analyses for each wave of T2–T6. The self-confidence data of each group are shown in Table A2. The results consistently showed no detectable moderating effect of self-confidence on CR reactivity in both Chinese Language and Mathematics tests at each wave of T2–T6 (see Table A3 for detailed results). Moreover, a set of six classes were randomly selected from the no-CR group to balance the sample sizes between the CR and no-CR groups, and the results showed the exact same patterns.

### 3.3. Multilevel Regression Analyses

Given the hierarchical data structure (that is, assessment waves nested within students, and students nested within classes), multilevel regression analyses were performed to further refine the analyses for CR reactivity and the moderating effect of self-confidence. Since the intraclass correlation (ICC) values for class clustering (i.e., Level 3) were below 0.05 (Peugh 2010), and the results exhibited consistent patterns across both the three-level and two-level regression models, we hence decided to report the results from the two-level models. For both Chinese Language and Mathematics subjects, we conducted multilevel regression analyses (Level 1: waves, Level 2: students) using the R *lme4* package (Bates et al. 2014). For each wave of T2–T6, the assessment scores for Chinese Language and Mathematics were standardized, respectively. The experimental group was dummy coded (CR group = 1, no-CR group = 0), self-confidence in each subject was mean-centered, and the assessment wave was also dummy coded (i.e., T2 as reference). A random intercept and a random slope of self-confidence were included; however, a random slope of self-confidence was excluded from the Chinese Language model because the model failed to converge.

As shown in Table 2, the results showed that children’s Chinese Language assessment scores could be predicted by their self-confidence in this domain, *β* = 0.01, *t* = 4.53, *p* < .001, 95% CI [0.01, 0.01], but not by group, *β* = −0.04, *t* = −0.61, *p* = .54, 95% CI [−0.19, 0.10], or the interaction between group and self-confidence, *β* = 0.001, *t* = 0.36, *p* = .72, 95% CI [−0.01, 0.01]. The model also found interaction effects between group and T5 (vs. T2) and between group and T6 (vs. T2), *p*s < .05 (see General Discussion for detailed discussion). However, the simple effects analysis indicated that there was no significant difference between the two groups at each of T2-T6, all *p*s > .05. Similarly, children’s Mathematics assessment scores could be predicted by their self-confidence in this domain, *β* = 0.03, *t* = 15.48, *p* < .001, 95% CI [0.02, 0.03], but not by group, *β* = 0.03, *t* = 0.51, *p* = .61, 95% CI [−0.10, 0.17], or the interaction between group and self-confidence, *β* = −0.001, *t* = −0.10, *p* = .92, 95% CI [−0.01, 0.01]. The model also found a significant interaction effect between group and T6 (vs. T2), *p* < .01 (see General Discussion for detailed discussion). However, the simple effects analysis indicated that there was no significant difference between the two groups at each wave of T2–T6, all *p*s > .05. As a summary, the multilevel regression results re-confirmed the absence of CR reactivity and the absence of the moderating role of self-confidence.

## 4. General Discussion

The current study is the first to (1) examine the potential reactive influence of CRs on elementary school children’s academic assessment performance in real educational settings, and (2) explore the potential moderating effect of self-confidence. The documented Bayesian evidence and results from multilevel regression analyses consistently suggest that soliciting CRs does not reactively alter children’s academic assessment performance in both Chinese Language and Mathematics tests, i.e., there is no reactivity effect of CRs on children’s academic performance in school settings. Furthermore, children’s self-confidence in performing academic exams does not moderate the reactivity effect. No prior research has examined whether instructing children to make concurrent CRs has a direct impact on their performing academic tests. This study utilized a large sample (*N* > 700) and employed a five-wave experimental design (from the middle grade to the end of primary school), providing exploratory yet reliable conclusions.

So far, there is not yet a clear consensus regarding CR reactivity. Although many studies found that retrospective CRs can reactively improve participants’ performance in cognitive tasks (e.g., Bonder and Gopher 2019; Double and Birney 2017, 2018, 2019a; Lei et al. 2020; Li et al. 2023), other studies showed that instructing participants to make CRs produces no direct influences on task performance (Ackerman and Goldsmith 2008; Ackerman 2014; Petrusic and Baranski 2003; Thompson et al. 2013).The general benefit hypothesis (Double and Birney 2017, 2019a) proposes that CRs may somehow increase task-related introspection, which in turn facilitate task performance. When participants are required to make CRs, in order to give an appropriate CR value, they must reflect on the correctness of the provided answer, and then report their subjective confidence level. The enhanced conservation theory (Li et al. 2023) suggests that the requirement of making CRs drives participants to be more careful in providing a response (that is, they need to gather more information before making a response). Hence, they are more likely to gain insights into the effectiveness of task strategies and make adjustments in subsequent test trials. In the field of JOL reactivity, the enhanced learning engagement theory (Shi et al. 2022; Zhao et al. 2022) assumes that positive reactivity results from enhanced learning engagement (e.g., study time, attention, and effort) introduced by the requirement of making JOLs. However, inconsistent with the predictions of the above theories, no positive reactivity effect of CRs was found in the current study.

Several possible explanations are available to account for the absence of the CR reactivity effect on children’s academic assessment performance. The first, and what we consider to be the main reason, is that children spontaneously monitor their on-line performance (e.g., error detection) in academic test situations. Specifically, completing academic tests is a regular activity for students in school, thus they may often conduct self-evaluations similar to confidence judgments to monitor whether they perform well in the exam. Therefore, asking participants to make CRs may produce minimal additional reflective influences compared to not making CRs. Many cross-sectional and longitudinal studies showed that children’s ability to realistically monitor their learning and memory increases during the primary school years (Roebers et al. 2019; Schneider and Löffler 2016). In late childhood, children can make relatively accurate metacognitive judgments on complex verbal tasks (e.g., Bayard et al. 2021; Steiner et al. 2020) and mathematics tasks (e.g., Baars et al. 2018; Rinne and Mazzocco 2014). Such findings were also documented in the current study (see Appendix A). Therefore, it may be that the benefits of making CRs, as suggested by the general benefit hypothesis (Double and Birney 2017, 2019a), can be realized by children themselves during academic test situations.

This explanation is consistent with evidence from other forms of metacognitive monitoring. For instance, think-aloud protocols (TAPs; Fox and Charness 2010; Leow and Morgan-Short 2004) measure metacognitive monitoring by concurrent verbalization (verbalizing the thoughts while performing a task). Previous studies found that think-aloud protocols can also reactively improve learning outcomes (Fox and Charness 2010). However, a meta-analysis of 94 studies showed that concurrent verbalization is not always reactive, unless it directs participants for additional processing, such as reflection or self-explanation (Bannert and Mengelkamp 2008; Fox et al. 2011). In a similar vein, recent researchers suggested that simply soliciting metacognitive judgments is necessary but not sufficient to induce reactivity, especially for educationally related materials (Davis and Chan 2023; Lee and Ha 2019). Recent evidence suggests that instructing students to make JOLs does not improve memory of educationally related texts (Ariel et al. 2021; Hausman and Kubik 2023) and general knowledge facts (Schäfer and Undorf 2023), and it does not provide additional benefits for retrieval practice (Zhao et al. 2023). However, asking students to make deep metacognitive reflections (e.g., providing four JOLs after reading each text section) yields positive reactivity effect on reading performance (Davis and Chan 2023).

A second possibility is that an academic assessment performance is highly valued by children themselves, and they typically have a strong intrinsic or extrinsic motivation to perform well in the exam, i.e., when taking academic tests, students are generally fully engaged regardless of whether they need to provide CRs or not. Hence, there is no further room left for CRs to enhance their engagement (Shi et al. 2022; Zhao et al. 2022), leading to a minimal reactivity of CRs on an academic assessment performance. Additionally, students may have been sufficiently careful in such important assessments. As a result, their awareness of searching for information relevant to the answer (Li et al. 2023) is no longer enhanced by the requirement of making CRs. In summary, eliciting CRs exerts little reactivity effect on children’s academic assessment performance.

In addition, other explanations should be considered. For instance, the learning and testing environments may play an important role. The majority of reactivity studies were conducted in laboratory settings using computer screens (Double and Birney 2018; Double et al. 2018; Li et al. 2023). Previous research indicated better performance on paper relative to screen-based tasks (i.e., screen inferiority; Sidi et al. 2017). Therefore, it is plausible that maximized task performance in paper-and-pencil contexts may limit the extent of metacognitive judgment reactivity. Furthermore, the time frames (i.e., 45 min) may restrict the depth of cognitive activities and metacognitive reflection. However, young children are shown to be capable of concurrently performing metacognitive judgment tasks (Koriat and Ackerman 2010; Roebers et al. 2019; Zhao et al. 2022).

The absence of the moderating role of self-confidence is inconsistent with the cognizant confidence hypothesis, which proposes that CRs prime a preexisting belief in one’s own abilities and thus have opposite effects on task performance between individuals with high versus low self-confidence. Two reasons may explain the results documented here. On the one hand, taking exams is an important part of their campus life. Children completed the standardized academic tests in the current study just like they took the midterm or final exams organized by school. Both the test content and the test format are familiar to them. Familiar tasks are less likely to invoke feelings of anxiety, nervousness, etc. (Ladd and Gabrieli 2015; Reeve et al. 2009). Therefore, participants’ a priori beliefs (i.e., self-confidence) activated by the requirement of providing CRs does not additionally modulate the reactivity effect. On the other hand, children are generally overconfident (García et al. 2016; Ots 2013; van Loon et al. 2017; Was and Al-Harthy 2018), which was also confirmed in the present study (see Appendix A), and thus may be less susceptible to confidence priming. Closely related to self-confidence, students’ self-concept or self-efficacy regarding their academic ability, which is formed through numerous past learning experiences, plays a stabilizing role in academic performance (Honicke and Broadbent 2016) and is unlikely to be additionally primed by the requirement of providing CRs.

Moreover, the present study also provided some insights into other potential moderators, namely, age, overall academic ability, and item difficulty. Zhao et al. (2022) found that the positive reactivity effect of JOLs tends to be small at Grades 1 and 3 but appreciably larger at Grade 5. Specifically, the proportions of participants who exhibited positive reactivity appreciably increased across grades. The current study found that the reactivity effect of CRs on children’s academic assessment performance did not change across T2–T6 (i.e., no reactivity in all five waves). Although the results from the multilevel regression analyses revealed significant interactions between group and T5 (vs. T2) and between group and T6 (vs. T2) in Chinese Language performance, and between group and T6 (vs. T2) in Mathematics performance, they all pertained to different directions of minor numerical group differences between T2 and the subsequent waves. A recent longitudinal study (three waves with six-month intervals) demonstrated that second graders’ cue utilization of CRs increased over time, while fourth graders did not show an increase in their cue utilization (Roebers et al. 2019). This may be related to development patterns found by Zhao et al. (2022) and the current study. Furthermore, students’ overall academic ability (i.e., average performance of both subjects across T1–T6) was included in the multilevel regression analyses. The results showed no significant interaction between academic ability and group in both domains, suggesting both high- and low-ability students show no reactivity effect in the testing situations of the current study.

Prior research found that the strength of cue-target relatedness moderates the JOL reactivity effect on the memory of word pairs, with a positive reactivity for related pairs and negative reactivity for unrelated pairs (Chang and Brainerd 2023; Li et al. 2024; Undorf et al. 2024). This suggests that the item difficulty may be a potential moderator of reactivity. Thus, we categorized each test into approximately equal numbers of difficult and easy items based on their difficulty levels (i.e., average correct answer rate), and then we included item difficulty (Difficult = 1, Easy = 0) in the multilevel regression analyses. For both subjects, the results showed no significant interactions between item difficulty and group, and the three-way interactions of item difficulty, group, and wave were also not significant. However, it is worth noting that the simple effects analyses revealed significant group differences in test scores on difficult items of the T6 Chinese Language test, *β* = 0.19, *t* = 2.44, *p* = .01, as well as significant group differences in test scores on difficult items of the T6 Mathematics test, *β* = −0.17, *t* = −2.20, *p* = .03. Namely, making CRs improved performance on difficult items of the T6 Chinese Language test, whereas it decreased performance on difficult items of the T6 Mathematics test. We propose that the observed pattern may be attributed to test difficulty and time constraints. As the Mathematics tests are quite challenging (e.g., average correct rate for T6 test: *M_difficult_* = 0.34, *SD_difficult_* = 0.11; *M_easy_* = 0.56, *SD_easy_* = 0.07), making CRs may guide students to save time spent on very difficult questions. Meanwhile, the Chinese tests are moderately difficult overall (e.g., average correct rate for T6 test: *M_difficult_* = 0.46, *SD_difficult_* = 0.10; *M_easy_* = 0.70, *SD_easy_* = 0.09); therefore, making CRs may encourage students to use their available time to reflect on moderately challenging questions. However, given that this result represents the only pattern observed across multiple assessment waves, we exercise caution regarding drawing conclusions from these findings.

When considering the moderating role of self-confidence, it may vary by subject. Chinese Language and Mathematics are two moderately related (*r* = .36) but distinct subjects (see a meta-analysis, Lu et al. 2022). Previous studies suggested that students were more likely to suffer from test anxiety in the Mathematics tests than in Language tests (Bong et al. 2012; Cassady and Johnson 2002). Hence, as inferred by the cognizant confidence hypothesis, the degree of difference between high and low self-confidence participants’ sense of affirming (or threatening) primed by making CRs may be greater in Mathematics tests than in the Language tests. However, the results of the current study run counter to this prediction by showing that self-confidence did not moderate the CR reactivity effect in both the Mathematics and Chinese Language tests.

It should be acknowledged that the current study suffers from several limitations. First, it is still premature to conclude no reactivity effect of CRs on children’s academic assessment performance in all subjects. Only two main subjects (i.e., Chinese Language and Mathematics) received investigation here. Whether children’ assessment performance in other academic domains (e.g., English Language, an important foreign language subject for Chinese elementary school students) is reactively affected by CRs remains unknown. It is also unclear whether performance in other tests that consist of questions evaluating simple knowledge (e.g., key-term definitions, translating English words, or reciting ancient poems), rather than the comprehensive tests as those in the current study, are affected by CRs. Future research is encouraged to explore the above two issues.

Overall, the current study provides consistent Bayesian evidence and multilevel regression results supporting the absence of a reactivity effect of CRs on elementary children’s academic assessment performance in real educational settings in both Chinese Language and Mathematics subjects. Furthermore, children’s self-confidence about their own ability to perform Chinese Language and Mathematics exams does not moderate the reactive influence of CRs. Future research to systematically explore these issues is called for.

## Figures and Tables

**Table 1:** Demographic information of the two groups.

Waves	CR Group			no-CR Group		
	*n*	Age	Gender	*n*	Age	Gender
T1	269	9.71 (0.31)	142 boys	522	9.72 (0.32)	275 boys
T2	269	10.21 (0.31)	142 boys	523	10.22 (0.32)	276 boys
T3	264	10.70 (0.31)	139 boys	517	10.71 (0.31)	271 boys
T4	262	11.21 (0.32)	138 boys	515	11.22 (0.31)	271 boys
T5	261	11.70 (0.31)	137 boys	511	11.72 (0.31)	269 boys
T6	261	12.21 (0.32)	136 boys	512	12.22 (0.31)	270 boys

Note. All participants did not receive experimental manipulation at T1. *SD* of the group mean is shown in parentheses.

**Table 2:** Results of the multilevel regression models for two subjects.

Fixed Effects	Chinese Language				Mathematics			
	*β*	*t*	*p*	95% CI	*β*	*t*	*p*	95% CI
Intercept	0.01	0.26	.80	[−0.07, 0.09]	−0.05	−1.38	.17	[−0.13, 0.02]
Group	−0.04	−0.61	.54	[−0.19, 0.10]	0.03	0.51	.61	[−0.10, 0.17]
Self-confidence	0.01	4.53	< .001	[0.01, 0.01]	0.03	15.48	<.001	[0.02, 0.03]
T3 (vs. T2)	−0.04	−1.42	.16	[−0.10, 0.02]	0.02	0.64	.52	[−0.05, 0.09]
T4 (vs. T2)	−0.04	−1.18	.24	[−0.10, 0.02]	0.02	0.59	.56	[−0.05, 0.09]
T5 (vs. T2)	−0.04	−1.23	.22	[−0.10, 0.02]	0.01	0.20	.84	[−0.06, 0.08]
T6 (vs. T2)	−0.05	−1.52	.13	[−0.11, 0.01]	0.05	1.35	.18	[−0.02, 0.12]
Group × Self-confidence	0.001	0.36	.72	[−0.01, 0.01]	−0.001	−0.10	.92	[−0.01, 0.01]
Group × T3 (vs. T2)	0.10	1.92	.05	[−0.002, 0.21]	−0.08	−1.34	.18	[−0.20, 0.04]
Group × T4 (vs. T2)	0.09	1.74	.08	[−0.01, 0.20]	−0.09	−1.43	.15	[−0.21, 0.03]
Group × T5 (vs. T2)	0.11	2.05	.04	[0.005, 0.21]	−0.08	−1.35	.18	[−0.20, 0.04]
Group × T6 (vs. T2)	0.14	2.69	.01	[0.04, 0.25]	−0.18	−2.87	<.01	[−0.29, −0.06]

## Data Availability

Given the data analyzed in the present study were collected by a large-scale longitudinal project conducted by many other researchers besides the current study’ authors, the data analyzed here hence have not been made publicly available. However, the authors agree to share the data with interested readers upon request.

## Note

1 There was a main effect of gender (boys = 1, girls = 0) on the average Chinese Language assessment scores, *b* = −3.46, *t* = −3.20, *p* < .001, with girls outperforming boys. There was no main effect of gender on the average Mathematics assessment scores, *b* = −1.18, *t* = −1.16, *p* = .25.

## Appendix A

For Chinese Language tests, participants in the CR group successfully provided item-by-item CRs to 98.82% (*SD* = 5.16%) of the test items at T2, 99.32% (*SD* = 2.35%) at T3, 99.06% (*SD* = 3.39%) at T4, 98.97% (*SD* = 6.54%) at T5, and 99.10% (*SD* = 6.31%) at T6. CRs were transferred from 1–7 to 0%−100% using the formula (CR−1)/6*100%. The average of transformed confidence was 77.88% (*SD* = 16.23%) at T2, 80.36% (*SD* = 18.78%) at T3, 81.06% (*SD* = 22.37%) at T4, 76.70% (*SD* = 21.54%) at T5, and 77.73% (*SD* = 23.18%) at T6. Considering the actual performance at each wave, participants consistently exhibited overconfidence from fourth to sixth grade. For each participant, a Gamma (*G*) correlation was calculated to quantify the relative accuracy of CRs. The *G*s from T2 to T6 were 0.49 (*SD* = 0.29), 0.47 (*SD* = 0.34), 0.34 (*SD* = 0.35), 0.40 (*SD* = 0.35), and 0.35 (*SD* = 0.43), respectively. The average of *G* values in each wave of T2–T6 was significantly greater than chance (0), *t*s ≥ 12.46, *p*s < .001, Cohen’s *d*s ≥ 0.82, all *BF*_10_ > 100, indicating that participants were able to distinguish correct from incorrect answers.

For Mathematics tests, participants in the CR group successfully provided item-by-item CRs to 98.56% (*SD* = 4.42%) of the test items at T2, 96.89% (*SD* = 7.26%) at T3, 96.35% (*SD* = 8.02%) at T4, 96.42% (*SD* = 5.55%) at T5, and 96.10% (*SD* = 7.35%) at T6. The average confidence (after scale transformation) was 75.87% (*SD* = 19.06%) at T2, 68.13% (*SD* = 23.57%) at T3, 71.22% (*SD* = 25.71%) at T4, 70.13% (*SD* = 24.59%) at T5, and 70.31% (*SD* = 25.63%) at T6, indicating that participants consistently overestimated their actual ability to perform Mathematics academic assessments. However, they adequately distinguished between correct and incorrect answers. From T2 to T6, the *G*s were 0.44 (*SD* = 0.41), 0.45 (*SD* = 0.38), 0.32 (*SD* = 0.47), 0.27 (*SD* = 0.51), and 0.21 (*SD* = 0.52), respectively, which were all significantly greater than 0, *t*s ≥ 6.39, *p*s < .001, Cohen’s *d*s ≥ 0.41, all *BF*_10_ > 100.

## Appendix B

Table A1 presents children’s academic assessment scores at each wave of T2–T6. For Chinese Language tests, the results showed no statistically detectable differences between the CR and no-CR groups in assessment scores at each wave. Similarly, for Mathematics assessment scores, no significant difference emerged between the two groups at each wave.

Table A2 lists children’s self-confidence in performing Chinese Language and Mathematics exams at each wave of T2–T6. Then, a series of multiple regression analyses were conducted on Chinese Language or Mathematics assessment scores for each wave of T2–T6. As shown in Table A3, the results consistently showed no detectable moderating effect of self-confidence on CR reactivity in both subjects at each wave of T2–T6.

**Table A1:** Independent sample *t*-test results of academic assessment scores at each wave.

Subjects	Waves	CR Group	no-CR Group	*t*	*p*	Cohen’s *d*	*BF*_10_
Chinese Language	T2	67.05 (16.17)	67.68 (13.81)	−0.57	.57	−0.04	0.10
	T3	70.10 (14.55)	68.89 (14.54)	1.10	.27	0.08	0.15
	T4	62.42 (17.05)	61.24 (17.52)	0.90	.37	0.07	0.13
	T5	55.71 (15.69)	54.41 (15.28)	1.11	.27	0.08	0.16
	T6	59.61 (16.53)	57.71 (17.39)	1.46	.15	0.11	0.24
Mathematics	T2	55.17 (16.47)	54.54 (15.37)	0.53	.60	0.04	0.10
	T3	40.36 (14.38)	41.04 (14.72)	−0.62	.54	−0.05	0.10
	T4	40.92 (16.37)	41.40 (16.61)	−0.38	.70	−0.03	0.09
	T5	43.40 (16.66)	43.58 (16.39)	−0.14	.89	−0.01	0.09
	T6	41.47 (20.71)	43.55 (21.32)	−1.29	.20	−0.10	0.19

**Table A2:** Independent sample *t*-test results of self-confidence scores at each wave.

Subjects	Waves	CR Group	no-CR Group	*t*	*p*	Cohen’s *d*	*BF*_10_
Chinese Language	T2	91.36 (9.31)	91.16 (7.81)	0.32	.75	0.02	0.09
	T3	90.73 (8.98)	90.43 (8.50)	0.46	.65	0.04	0.09
	T4	92.31 (7.68)	91.55 (7.70)	1.30	.19	0.10	0.20
	T5	92.10 (6.82)	90.92 (7.12)	2.20	.03	0.17	0.90
	T6	92.08 (8.36)	92.10 (6.79)	−0.04	.97	−0.003	0.09
Mathematics	T2	91.04 (10.94)	91.41 (8.85)	−0.51	.61	−0.04	0.10
	T3	89.60 (11.51)	90.43 (9.62)	−1.06	.29	−0.08	0.15
	T4	89.96 (10.02)	89.59 (9.99)	0.49	.63	0.04	0.10
	T5	89.42 (11.45)	88.60 (10.81)	0.97	.33	0.07	0.14
	T6	88.63 (11.68)	88.37 (10.51)	0.32	.75	0.02	0.09

**Table A3:** Regression model results about the moderating effect of self-confidence at each wave.

Subjects	Chinese Language				Mathematics			
	*β*	*t*	*p*	*BF*_incl_	*β*	*t*	*p*	*BF*_incl_
T2 regression model	*R*^2^ = .18 ***				*R*^2^ = .24 ***			
Group	−1.07	−1.08	.28	0.10	0.91	0.88	.38	0.08
Self-confidence	0.75	9.91	<.001	>100	0.84	12.28	<.001	>100
Group × Self-confidence	0.07	0.57	.57	0.05	−0.10	−1.01	.31	0.05
T3 regression model	*R*^2^ = .16 ***				*R*^2^ = .25 ***			
Group	1.12	1.10	.27	0.11	0.06	0.06	.95	0.05
Self-confidence	0.64	9.22	<.001	>100	0.73	12.59	<.001	>100
Group × Self-confidence	0.05	0.45	.65	0.06	−0.08	−0.83	.41	0.03
T4 regression model	*R*^2^ = .16 ***				*R*^2^ = .20 ***			
Group	0.33	0.27	.79	0.07	−0.58	−0.51	.61	0.06
Self-confidence	0.83	9.07	<.001	> 100	0.71	10.68	<.001	>100
Group × Self-confidence	0.24	1.42	.16	0.09	0.08	0.72	.47	0.04
T5 regression model	*R*^2^ = .11 ***				*R*^2^ = .22 ***			
Group	0.16	0.15	.88	0.07	−0.83	−0.75	.46	0.07
Self-confidence	0.69	7.55	<.001	>100	0.73	12.07	<.001	>100
Group × Self-confidence	0.12	0.76	.45	0.05	−0.08	−0.83	.41	0.05
T6 regression model	*R*^2^ = .09 ***				*R*^2^ = .23 ***			
Group	1.91	1.53	.13	0.26	−2.70	−1.90	.06	0.35
Self-confidence	0.58	5.42	<.001	>100	0.99	12.51	<.001	>100
Group × Self-confidence	0.22	1.36	.18	0.28	−0.18	−1.40	.16	0.28

*Note*. *** *p* < .001.
